# Sensitive Detection and Differentiation of Biologically Active Ricin and Abrin in Complex Matrices via Specific Neutralizing Antibody-Based Cytotoxicity Assay

**DOI:** 10.3390/toxins16060237

**Published:** 2024-05-23

**Authors:** Zhi Li, Bo Ma, Mengqiang Gong, Lei Guo, Lili Wang, Hua Xu, Jianwei Xie

**Affiliations:** Laboratory of Toxicant Analysis, Academy of Military Medical Sciences, Beijing 100850, China

**Keywords:** ricin, abrin, activity, neutralizing antibody, cytotoxicity assay, complex matrices

## Abstract

Ricin and abrin are highly potent plant-derived toxins, categorized as type II ribosome-inactivating proteins. High toxicity, accessibility, and the lack of effective countermeasures make them potential agents in bioterrorism and biowarfare, posing significant threats to public safety. Despite the existence of many effective analytical strategies for detecting these two lethal toxins, current methods are often hindered by limitations such as insufficient sensitivity, complex sample preparation, and most importantly, the inability to distinguish between biologically active and inactive toxin. In this study, a cytotoxicity assay was developed to detect active ricin and abrin based on their potent cell-killing capability. Among nine human cell lines derived from various organs, HeLa cells exhibited exceptional sensitivity, with limits of detection reaching 0.3 ng/mL and 0.03 ng/mL for ricin and abrin, respectively. Subsequently, toxin-specific neutralizing monoclonal antibodies MIL50 and 10D8 were used to facilitate the precise identification and differentiation of ricin and abrin. The method provides straightforward and sensitive detection in complex matrices including milk, plasma, coffee, orange juice, and tea via a simple serial-dilution procedure without any complex purification and enrichment steps. Furthermore, this assay was successfully applied in the unambiguous identification of active ricin and abrin in samples from OPCW biotoxin exercises.

## 1. Introduction

Ricin and abrin, two of the most lethal plant toxins, are mainly derived from the seed of *Ricinus communis* and *Abrus precatorius*, respectively. Both of them belong to the family of type II ribosome-inactivating proteins (RIPs) with high structural similarity and sequence homology. Ricin is the only biotoxin covered by the Chemical Weapons Convention (CWC) as well as the Biological and Toxin Weapons Convention (BTWC). In addition, both ricin and abrin are categorized as category B biothreat agents by the USA Center for Disease Control and Prevention (CDC) [1,2,3]. Their high toxicity, easy availability, and lack of effective antidotes make them potential bioweapons and bioterrorism agents, underscoring the critical need for convenient and sensitive detection methods.

A variety of analytical techniques have been developed for detecting ricin and abrin, based on their intrinsic physicochemical and immunological properties [4,5,6]. Mass spectrometric approaches can provide sequence and structural information achieved through the accurate identification of unique peptides after enzymatic digestion, which are necessary for the unambiguous confirmation of the toxins [7,8]. Nonetheless, these methods are usually time-consuming, costly, not sufficiently sensitive, susceptible to matrix interference, and require sophisticated extraction and enrichment procedures and professional personnel. Immunological methods, such as enzyme-linked immunosorbent assay (ELISA) and lateral flow assay (LFA), are favored for their simplicity, high sensitivity, and efficiency [9,10,11]. However, a major disadvantage of these methods is the inability to distinguish between biologically active and inactive toxin. The discrimination of functionally active or inactive toxin is extremely important to determine the risk level and appropriate management of a suspected toxin exposure incident, because the inactivated toxin no longer poses a threat [12]. Both ricin and abrin are composed of a catalytically active A chain and a lectin B chain, which are linked by a single disulfide bond. It is known that the A chain with *N*-glycosidase activity (EC 3.2.2.22) is required for ribosome inactivation through depurinating the 28S rRNA of the 60S ribosomal subunit and thereby inhibiting protein synthesis. The galactose-specific B chain is necessary for binding, internalization, and trafficking of the toxin into cells [13]. Generally, the depurination activity of the A chain can be detected via adenine-release assay or rabbit reticulocyte lysate translation assay [14,15]. The enzyme-linked lectin assay determines the glycan binding activity of the B chain [16]. However, activity detection of the separated chain only provides limited information on the toxicity of the intact toxin, because neither the A chain nor the B chain are significantly toxic in isolation. Only intact and active ricin and abrin have sufficiently strong potency to induce cell death in various cell lines. Consequently, cell-based cytotoxicity assays emerge as a promising strategy, offering a clear advantage in displaying the toxin’s full biological activity. Currently, cytotoxicity can be detected using different endpoint measurements, such as colorimetry, fluorescence, or luminescence, or a real-time assay based on an impedance sensor technique to monitor cell viability and proliferation without labeling [17,18,19,20]. However, the sensitivity of a cytotoxicity assay is directly influenced by the choice of cell line and its susceptibility to the toxin [21,22]. In addition, most assays lack specific functional neutralizing antibodies to exclude cytotoxicity caused by irrelevant matrix components or to differentiate the cytotoxicity from other RIP toxins, particularly in the case of abrin detection [23,24,25].

In this study, we selected nine human cell lines, each originating from distinct organs, to assess the sensitivity of the cytotoxicity assay. Among all these cell lines, HeLa cells were found to be the most sensitive to both ricin and abrin, with the lowest IC_50_ values (1.18 and 0.14 ng/mL) and limits of detection (LODs) (0.3 and 0.03 ng/mL). High sensitivity, good robustness, rapid proliferative capacity, and ease of cultivation make HeLa cells an optimal choice for toxin detection. Using their specific functional blocking antibodies, it is feasible to differentiate ricin and abrin and exclude cytotoxicity caused by other toxic compounds. Moreover, this assay is capable of detecting both toxins in a variety of complex matrices such as plasma, milk, coffee, orange juice, and tea through a simple serial-dilution procedure without any complex purification and enrichment steps. Furthermore, the developed method was successfully applied for the sensitive and effective identification and differentiation of active ricin and abrin from unknown samples of biotoxin exercises conducted by the Organization for the Prohibition of Chemical Weapons (OPCW).

## 2. Results and Discussion

### 2.1. HeLa Cells Exhibited the Highest Sensitivity to Both Ricin and Abrin Exposures

**Figure 1:**
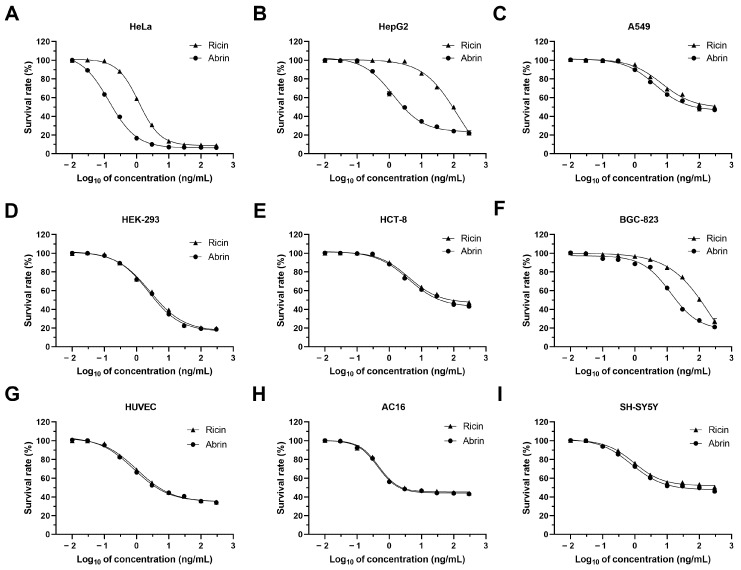
Survival curves of nine human-derived cell lines upon exposure to ricin and abrin. HeLa (**A**), HepG2 (**B**), A549 (**C**), HEK-293 (**D**), HCT-8 (**E**), BGC-823 (**F**), HUVEC (**G**), AC16 (**H**), and SH-SY5Y (**I**) cells were seeded into 96-well cell culture plates at a density of 1.0 × 10^4^ cells per well and were then exposed to a range of ricin and abrin concentrations, from 0.01 to 300 ng/mL, for 24 h. Cell viability was assessed and survival curves were generated using the CCK-8 assay.

Based on the main target organs injured through ricin and abrin intoxication, nine human cell lines were exposed to the two toxins at concentrations ranging from 0.01 to 300 ng/mL for the evaluation of cytotoxicity and selection of the cell line with highest sensitivity. The selected cell lines were HeLa, HepG2, A549, HEK-293, HCT-8, BGC-823, HUVEC, AC16, and SH-SY5Y, derived from the cervix, liver, lung, kidney, intestine, stomach, endothelium, heart, and nervous system, respectively. Our findings indicated significant variability in the cytotoxicity of ricin and abrin across different cell lines. HeLa cells were found to be the most sensitive to both ricin and abrin, achieving the lowest IC_50_ values (the concentrations required for a 50% reduction in cell survival) of 1.18 and 0.14 ng/mL, with corresponding LODs at 0.3 and 0.03 ng/mL (Figure 1, Table 1). A549, HCT-8, HUVEC, AC16, and SH-SY5Y cells displayed resistance to cell death, with no significant increase in cell mortality observed upon elevating the toxin concentration to 300 ng/mL.

**Figure 2:**
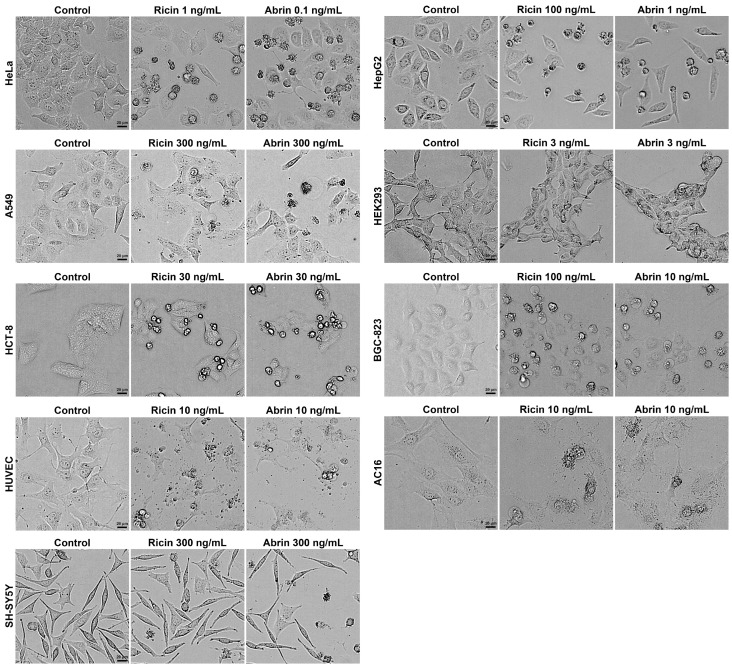
Different cell lines were seeded into 96-well cell culture plates at a density of 1.0 × 10^4^ cells per well and exposed to ricin and abrin at indicated concentrations for 24 h. Cellular morphology was visualized and recorded using a high-content cell analysis system in bright-field mode. Scale bars: 20 μm.

Upon exposure, the majority of cell lines exhibited the classic morphological hallmarks of apoptosis, characterized by cell shrinkage, membrane blebbing, and the formation of apoptotic bodies (Figure 2). Previous studies have reported that ricin and abrin can induce apoptosis through caspase activation, suggesting that the observed morphological changes in various cell lines in the present study were likely to have been due to apoptosis [26,27]. We propose that the variable cytotoxicity observed across different cell lines may stem from disparities in the quantity of glycan-binding sites on the cell membrane or variations in the toxins’ cell binding affinity. Such differences could affect the internalization and intracellular amounts of toxins, leading to varied cytotoxic outcomes [21,28,29,30]. Additionally, the different expression levels of key regulatory proteins in apoptotic signaling pathways among various cell lines may be another possible reason [22]. Notably, a minority of the cell lines, including HeLa, HepG2, and BGC-823, showed markedly different IC_50_ values for ricin and abrin. The mechanisms underlying this phenomenon require further investigation. Elucidating these mechanisms may be essential for the identification of more specific toxic effects and the development of cell lines with even greater sensitivity. Given its exceptional sensitivity, good robustness, rapid proliferative capacity, and ease of cultivation, the HeLa cell line was selected as the optimal model for toxin detection in subsequent experiments.

### 2.2. Optimization of Neutralizing Antibodies Concentrations for Ricin and Abrin-Specific Detection

**Figure 3:**
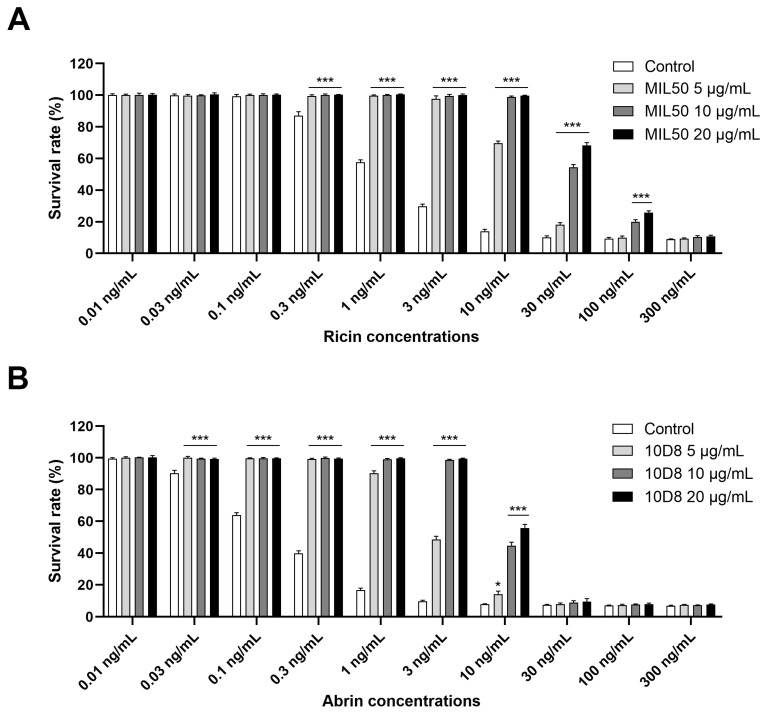
Optimization of neutralizing antibody concentrations for ricin and abrin-specific detection. Ricin (**A**) and abrin (**B**) at concentrations ranging from 0.01 to 300 ng/mL were freshly prepared in serum-free medium and preincubated with mAbs MIL50 or 10D8, respectively, at final concentrations of 5, 10, and 20 μg/mL for 1 h at 37 °C. Toxins with or without antibody pretreatment were added to HeLa cells cultured in 96-well plates at a density of 1.0 × 10^4^ cells per well and incubated for 24 h at 37 °C. The cell survival rate was assessed using CCK-8 assay, with data presented as the mean ± SD (*n* = 3). Statistical significance was evaluated using one-way ANOVA (* *p* < 0.05, *** *p* < 0.001 vs. control).

A distinct advantage of the cytotoxicity assay is its ability to measure the full biological activity of intact toxin. Furthermore, the assay is also high-throughput and highly sensitive. However, the assay’s selectivity and specificity may be compromised by similar cytotoxic effects of other toxic agents. To address this problem, pre-incubation of the sample with toxin-specific blocking antibodies was essential to exclude cytotoxicity induced by irrelevant compounds and make a distinction between ricin and abrin. HeLa cells were subjected to different concentrations of ricin and abrin preincubated with or without monoclonal antibodies (mAbs) MIL50 or 10D8 at final concentrations of 5, 10, and 20 μg/mL for 1 h at 37 °C. Results demonstrated that MIL50 or 10D8 treatment significantly inhibited cytotoxicity induced by ricin or abrin. Specifically, MIL50 at 5 or 10 μg/mL completely abolished cytotoxicity induced by ricin at concentrations up to 3 or 10 ng/mL, respectively. However, at concentrations exceeding 30 ng/mL, increasing MIL50 concentration to 20 μg/mL provided only marginal additional protection against cytotoxicity (Figure 3A). Similarly, 10D8 at 5 or 10 μg/mL fully prevented cytotoxicity caused by abrin at concentrations below 1 or 3 ng/mL, respectively, with limited improvement observed at 20 μg/mL for abrin at concentrations exceeding 10 ng/mL (Figure 3B). Based on these results, a neutralizing antibody concentration of 10 μg/mL was determined to be optimal and used in subsequent experiments.

### 2.3. Assessment of Neutralizing Antibodies’ Cross-Reactivity

**Figure 4:**
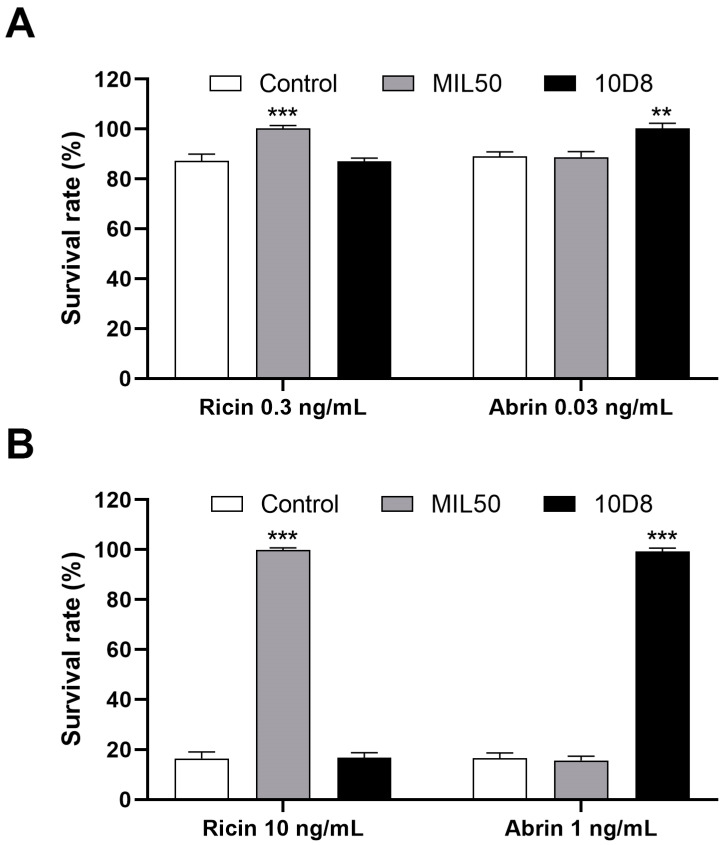
Assessment of neutralizing antibodies’ cross-reactivity. Low (**A**) and high (**B**) concentrations of ricin and abrin were treated with both MIL50 and 10D8 at a final concentration of 10 μg/mL for 1 h at 37 °C, then added to HeLa cells and incubated for 24 h at 37 °C. The survival rate of cells was calculated via CCK-8 assay. Data are presented as the mean ± SD (*n* = 3). Statistical significance was evaluated using one-way ANOVA (** *p* < 0.01, *** *p* < 0.001 vs. control).

The specificity of neutralizing antibodies is crucial for the accurate identification and differentiation of toxins. To determine the specificity and cross-reactivity of mAbs, ricin and abrin at indicated concentrations were pre-treated with both MIL50 and 10D8 at a final concentration of 10 μg/mL. Results showed that pretreatment with MIL50 effectively neutralized cytotoxicity induced by different concentrations of ricin. In contrast, 10D8 did not exhibit any protective effect against ricin’s cytotoxicity. Conversely, 10D8 pretreatment was able to eliminate cytotoxicity caused by abrin exposure, whereas MIL50 had no such effect (Figure 4). These results underscore the high specificity of the two mAbs. Consequently, the assay described herein was able to reliably distinguish between ricin and abrin using their respective blocking mAbs.

### 2.4. Impact of Complex Matrices on Cell Growth and Toxin Detection

**Figure 5:**
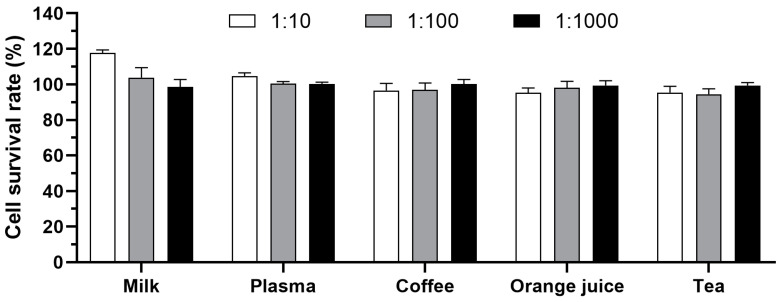
Impact of complex matrices on cell growth. Milk, plasma, coffee, orange juice, and tea were serially diluted to 1:10, 1:100, and 1:1000 with serum-free RPMI-1640 medium. The diluted samples were centrifuged, filtered, added to HeLa cells, and incubated for 24 h at 37 °C. The survival rate of cells was calculated via CCK-8 assay.

Firstly, the impact of various complex matrices on cell growth were assessed through serially diluting plasma, milk, orange juice, green tea, and coffee with serum-free RPMI-1640 medium. Notably, milk at a 1:10 dilution slightly promoted cell growth, whereas other matrices exhibited no significant impact (Figure 5). An appropriate dilution ratio is crucial to minimize matrix effects on cell growth, facilitating the accurate and reliable detection of toxins. Based on these findings, it is feasible to detect toxin samples in complex matrices by adjusting the dilution to a range of 1:10 to 1:1000 for subsequent analysis.

**Figure 6:**
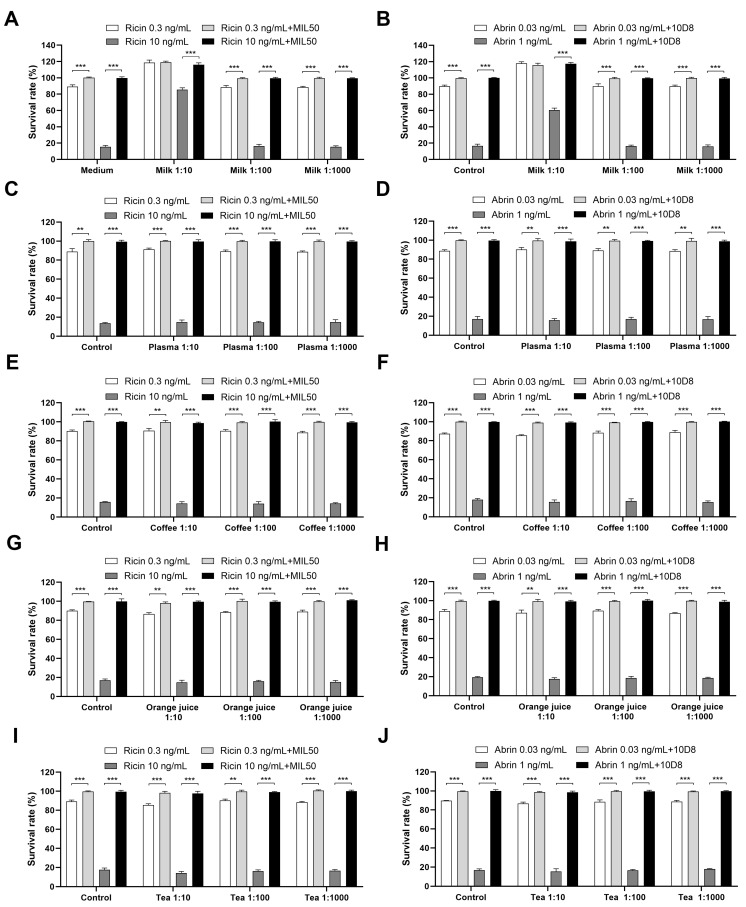
Impact of complex matrices on toxin detection. Ricin and abrin at indicated concentrations were spiked into the diluted milk (**A**,**B**), plasma (**C**,**D**), coffee (**E**,**F**), orange juice (**G**,**H**), and tea (**I**,**J**). The samples were preincubated with or without mAbs MIL50 or 10D8, respectively, at a final concentration of 10 μg/mL for 1 h at 37 °C, added to HeLa cells, and incubated for 24 h at 37 °C. The cell survival rates were calculated via CCK-8 assay. Data are presented as the mean ± SD (*n* = 3). Statistical significance was determined using Student’s *t* test (** *p* < 0.01, *** *p* < 0.001).

Next, the impact of various complex matrices on toxin detection was assessed through spiking ricin and abrin into the diluted matrices, with or without pre-treatment with mAbs MIL50 or 10D8. Notably, the findings revealed that a 1:10 diluted milk matrix significantly reduced the cytotoxicity of ricin or abrin by approximately 30- or 10-fold, respectively (Figure 6A,B). However, no significant effects were observed with other matrices. The observed reduction in cytotoxicity may be attributed to the interaction between the galactose present in milk and the toxin, which could have potentially interfered with and inhibited the toxin’s ability to bind to cells [31,32,33,34]. This result is important as it highlights the need for appropriate dilution for detecting toxins in milk samples. If the milk is diluted 1:10, ricin and abrin can be detected down to 10 ng/mL and 0.3 ng/mL, corresponding to concentrations of 100 ng/mL and 3 ng/mL in undiluted milk. Meanwhile, a 1:100 dilution did not interfere with the assay, allowing detection down to 0.3 ng/mL and 0.03 ng/mL, corresponding to 30 ng/mL and 3 ng/mL in the original milk sample (Figure 6A,B). Consequently, for the detection of toxins in milk samples, a 100-fold dilution is recommended to mitigate matrix interference and enhance assay sensitivity. For other complex matrices like plasma, tea, orange juice, and coffee, a 10-fold dilution is sufficient for sensitive toxin detection (Figure 6C–J). In summary, the developed assay provided LODs of 30 ng/mL for ricin and 3 ng/mL for abrin in milk, and 3 ng/mL and 0.3 ng/mL in other matrices including plasma, tea, orange juice, and coffee. The established detection limits for ricin and abrin in complex matrices provide valuable data for future reference and comparison.

### 2.5. Detection of Ricin and Abrin from the Samples of OPCW Biotoxin Exercises

**Figure 7:**
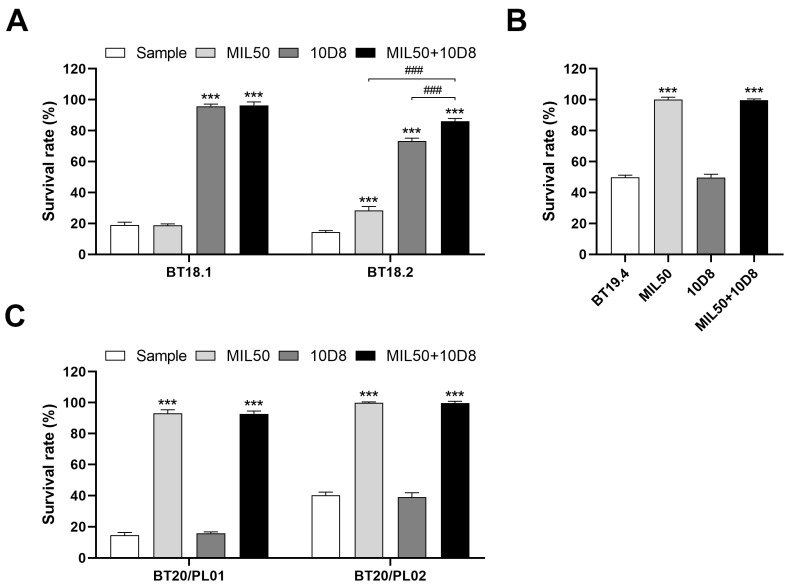
Detection of ricin and abrin from the samples of OPCW biotoxin exercises. (**A**) A 20 mg aliquot of each sample was dissolved in 1 mL PBS buffer, vortexed, and centrifuged. The resulting supernatant was subsequently diluted to a final ratio of 1:1000 with serum-free RPMI-1640 medium. (**B**) Spray buffer sample BT19.4 was diluted to a final ratio of 1:20,000 with serum-free RPMI-1640 medium. (**C**) Human plasma samples BT20/PL01 and BT20/PL02 were prepared via dilution to a final ratio of 1:1000 in serum-free RPMI-1640 medium. The diluted samples were preincubated with mAbs MIL50 or 10D8, either individually or in combination, at a final concentration of 10 μg/mL for 1 h at 37 °C and then added to cells. The cell survival rate was calculated via CCK-8 assay. Data are shown as the mean ± SD (*n* = 3). Statistical significance was determined using one-way ANOVA (*** *p* < 0.001 vs. sample, ^###^ *p* < 0.001).

The developed method was successfully used for the unambiguous identification of ricin and abrin in unknown samples from OPCW biotoxin exercises (Table 2). Specifically, during the third biotoxin exercise, the method effectively detected ricin and abrin spiked into protein powder samples, utilizing a 1:1000 dilution ratio after dissolving for optimal sensitivity. For sample BT18.2, abrus agglutinin rather than purified abrin was spiked into the protein powder. It is important to note that abrus agglutinin is a 40% ammonium sulfate precipitate from the preparation of abrin. Consequently, the sample was expected to contain abrin. However, the accurate concentration of abrin within this sample was presumed to be significantly lower than that of the abrus agglutinin present. Based on our results, treatment with either MIL50 or 10D8 antibodies individually resulted in the inhibition of cytotoxicity. Moreover, simultaneous application of both MIL50 and 10D8 antibodies led to a synergistic effect and further increased cell survival. Given these neutralization profiles, it is evident that sample BT18.2 contained both ricin and abrin toxins (Figure 7A). In the sample from the fourth biotoxin exercise, ricin was spiked into spray buffer, which consisted of PBS with Tween-20 (PBST) and various organic solvents (including polypropyleneglycol monobutylether, PEG200, glycerol, ethanol, and isopropanol), at a concentration of 30 μg/mL. Notably, the spray buffer itself exhibited significant cytotoxicity at dilution ratios below 1:5000, potentially interfering with the detection of ricin. However, the challenge was overcome and ricin was still sensitively detected at a 1:20,000 dilution ratio, where the cytotoxicity caused by the spray buffer was no longer apparent (Figure 7B). In the fifth biotoxin exercise, all ricin samples spiked into human plasma at concentrations varying from 1 to 16 μg/mL were successfully detected using a 1:1000 dilution ratio (Figure 7C). These results further demonstrate the method’s robustness and reliability in detecting real samples in the presence of complex matrices. Future work should focus on expanding the assay’s applicability to other toxins and further improving its robustness against the challenges posed by various complex matrices.

## 3. Conclusions

The present study successfully developed a sensitive, convenient, and high-throughput cytotoxicity assay for the efficient detection and differentiation of active ricin and abrin in complex matrices. The assay employs a straightforward serial-dilution procedure, circumventing the need for laborious purification or enrichment processes. The current method could be extended to a range of other non-cytotoxic or cytotoxic matrices with appropriate dilution ratios, providing a valuable tool for the detection and identification of ricin and abrin in diverse and challenging environments.

## 4. Materials and Methods

### 4.1. Materials

Ricin and abrin were prepared in house and purified to an estimated purity higher than 95% as described previously [35,36,37]. All toxins were handled by well-trained personnel wearing appropriate protective equipment in a clean fume hood. Toxin-containing solutions and consumable materials were decontaminated with sodium hydroxide or inactivated through autoclaving. Ricin-specific neutralizing monoclonal antibody (mAb) MIL50 was provided by Prof. Jiannan Feng’s team at the Academy of Military Medical Sciences (China). Abrin-specific neutralizing mAb 10D8 was prepared in our previous research [38].

### 4.2. Cell Culture

HepG2, HeLa, HCT-8, and BGC-823 cells were maintained in Roswell Park Memorial Institute 1640 medium (RPMI-1640, Gibco, Grand Island, NY, USA). HEK-293 and SH-SY5Y cells were propagated in minimum essential medium (MEM, Sigma-Aldrich, St. Louis, MO, USA). A549 cells were cultured in F-12K Nutrient Mixture (Gibco, Grand Island, NY, USA). HUVEC and AC16 cells were grown in high-glucose Dulbecco’s modified Eagle’s medium (DMEM, Gibco, Grand Island, NY, USA). Each medium was supplemented with 10% fetal bovine serum, 100 units/mL penicillin, and 100 µg/mL streptomycin (Gibco, Grand Island, NY, USA). The cells were incubated at 37 °C in an atmosphere containing 5% CO_2_ in a humidified incubator.

### 4.3. Cytotoxicity Evaluation

Cells were cultured in 96-well plates at a density of 1.0 × 10^4^ cells per well. Ricin and abrin, which were freshly prepared in sterile PBS, were serially diluted with serum-free medium to concentrations ranging from 0.01 to 300 ng/mL and introduced to each well after the removal of serum-containing growth media. Following a 24 h incubation at 37 °C, cell morphology was visualized and recorded using a high-content cell analysis system (Opera Phenix, PerkinElmer, Waltham, MA, USA) in bright-field mode. CCK-8 reagent (Beyotime, Shanghai, China) was added into each well and incubated for an additional hour at 37 °C. Absorbance at 450 nm was measured using a microplate reader (Infinite M1000 pro, TECAN, Männedorf, Switzerland). The cell survival rate was calculated using the following formula: survival rate % = (A450 _sample_ − A450 _background_/A450 _control_ − A450 _background_) × 100%. The survival curve was derived from the “log(inhibitor) vs. response-viable slope (four parameters)” nonlinear regression model, represented by the equation: Y = Bottom + (Top − Bottom)/(1 + 10 ^((LogIC50 − X) * HillSlope)^). IC_50_ value (the concentration required for a 50% reduction in cell survival) was determined through interpolation from the equation using the “interpolate unknowns from standard curve” function in GraphPad Prism with a Y value set to 50%. The limits of detection (LODs) were determined based on the lowest concentrations of toxin used in the experiments that produced a significant cytotoxic effect.

### 4.4. Antibody Treatment

Ricin and abrin at concentrations ranging from 0.01 to 300 ng/mL were freshly prepared in serum-free medium and preincubated with or without mAbs MIL50 or 10D8, respectively, at final concentrations of 5, 10, and 20 μg/mL for 1 h at 37 °C. Alternatively, for the evaluation and determination of cross-reactivity of mAbs, ricin and abrin were treated with both MIL50 and 10D8 at a final concentration of 10 μg/mL for 1 h at 37 °C. Toxins with or without antibody pretreatment were added to HeLa cells cultured in 96-well plates at a density of 1.0 × 10^4^ cells per well and incubated for 24 h at 37 °C. CCK-8 reagent was added into each well and incubated for an additional hour at 37 °C. Absorbance at 450 nm was measured and the cell survival rate was calculated.

### 4.5. Analysis of Complex Matrix Samples

The matrices used in this study were whole milk (Yili, Hohhot, China), NFC (not from concentrate) orange juice (Nongfu Spring, Hangzhou, China), Oriental Leaf green tea (Nongfu Spring, Hangzhou, China), and TANBING no sugar black coffee beverage (Nongfu Spring, Hangzhou, China). To determine the impact of complex matrices on cell growth, plasma, milk, orange juice, green tea, and coffee were serially diluted to 1:10, 1:100, and 1:1000 with serum-free RPMI-1640 medium. The diluted samples were centrifuged, filtered through a 0.22 μm filter, and then added to cells grown in 96-well cell culture plates at a density of 1.0 × 10^4^ cells per well. For the analysis of toxin-spiked complex matrix samples, ricin and abrin were spiked into the diluted matrices at the indicated concentrations. The samples were preincubated with or without mAbs MIL50 or 10D8, respectively, at a final concentration of 10 μg/mL for 1 h at 37 °C and then added to cells. After 24 h incubation at 37 °C, CCK-8 reagent was introduced into each well and incubated for an additional hour at 37 °C. Absorbance at 450 nm was measured and the cell survival rate was calculated.

### 4.6. Analysis of OPCW Biotoxin Exercise Samples

For protein powder samples BT18.1 and BT18.2, obtained from the third OPCW biotoxin exercise, a 20 mg aliquot of each sample was dissolved in 1 mL PBS buffer, vortexed, and centrifuged. The resulting supernatant was subsequently diluted to a final ratio of 1:1000 with serum-free RPMI-1640 medium. The spray buffer sample BT19.4, derived from the fourth OPCW biotoxin exercise, was diluted to a final ratio of 1:20,000 with serum-free RPMI-1640 medium. Human plasma samples BT20/PL01 and BT20/PL02, from the fifth OPCW biotoxin exercise, were prepared through dilution to a final ratio of 1:1000 in serum-free RPMI-1640 medium. The diluted samples were preincubated with mAbs MIL50 or 10D8, either individually or in combination, at a final concentration of 10 μg/mL for 1 h at 37 °C and then added to cells. After 24 h incubation at 37 °C, CCK-8 reagent was added into each well and incubated at 37 °C for 1 h. Absorbance at 450 nm was measured and the survival rate of cells was calculated.

### 4.7. Statistical Analysis

Data are presented as the mean ± standard deviation (SD) from three independent experimental replicates. Statistical analyses were conducted using Student’s *t* test for two-group comparisons or one-way analysis of variance (ANOVA) followed by Tukey’s multiple comparisons test with GraphPad Prism 8 software (* *p* < 0.05, ** *p* < 0.01, *** *p* < 0.001).

## Figures and Tables

**Table 1:** The IC_50_ values and LODs for ricin and abrin in nine human-derived cell lines.

Cell Type	Ricin IC_50_ (ng/mL)	Ricin LOD (ng/mL)	Abrin IC_50_ (ng/mL)	Abrin LOD (ng/mL)
HeLa	1.18	0.30	0.14	0.03
HepG2	130.20	10.00	1.30	0.30
A549	>300.00	3.00	67.61	1.00
HEK293	2.57	0.30	2.34	0.30
HCT-8	74.99	1.00	36.52	1.00
BGC-823	250.40	10.00	12.80	1.00
HUVEC	5.01	0.30	4.22	0.30
AC16	3.16	0.30	2.70	0.30
SH-SY5Y	>300.00	0.30	42.17	0.30

**Table 2:** The detection results of samples from OPCW biotoxin analysis exercises.

No.	Sample Name	Spiking Chemicals (Concentration)	Matrices	Dilution Ratio	Detection Results
3rd	BT18.1	Abrin (150 μg/g)	Protein powder	1:1000	Abrin
	BT18.2	Ricin (15 μg/g) Abrus agglutinin (300 μg/g) *	Protein powder	1:1000	Ricin Abrin
4th	BT19.4	Ricin (30 μg/mL)	Spray buffer	1:20,000	Ricin
5th	BT20/PL01	Ricin (16 μg/mL)	Human plasma	1:1000	Ricin
	BT20/PL02	Ricin (1 μg/mL)	Human plasma	1:1000	Ricin

* Abrus agglutinin is the 40% ammonium sulfate precipitate from the same preparation and contains abrin.

## Data Availability

Data are contained within the article and further inquiries can be directed to the corresponding authors.

## References

[1] Felder E., Mossbrugger I., Lange M., Wolfel R. (2012). Simultaneous detection of ricin and abrin DNA by real-time PCR (qPCR). Toxins.

[2] Stirpe F., Battelli M.G. (2006). Ribosome-inactivating proteins: Progress and problems. Cell Mol. Life Sci..

[3] Janik E., Ceremuga M., Saluk-Bijak J., Bijak M. (2019). Biological Toxins as the Potential Tools for Bioterrorism. Int. J. Mol. Sci..

[4] Bozza W.P., Tolleson W.H., Rivera Rosado L.A., Zhang B. (2015). Ricin detection: Tracking active toxin. Biotechnol. Adv..

[5] Dorner B.G., Zeleny R., Harju K., Hennekinne J.-A., Vanninen P., Schimmel H., Rummel A. (2016). Biological toxins of potential bioterrorism risk: Current status of detection and identification technology. TrAC Trends Anal. Chem..

[6] Robb C.S. (2015). The analysis of abrin in food and beverages. TrAC Trends Anal. Chem..

[7] Kalb S.R., Schieltz D.M., Becher F., Astot C., Fredriksson S.A., Barr J.R. (2015). Recommended Mass Spectrometry-Based Strategies to Identify Ricin-Containing Samples. Toxins.

[8] Liang L.H., Yang Y., Geng S., Cheng X., Yu H.L., Liu C.C., Liu S.L. (2021). Rapid Differential Detection of Abrin Isoforms by an Acetonitrile- and Ultrasound-Assisted On-Bead Trypsin Digestion Coupled with LC-MS/MS Analysis. Toxins.

[9] Simon S., Worbs S., Avondet M.A., Tracz D.M., Dano J., Schmidt L., Volland H., Dorner B.G., Corbett C.R. (2015). Recommended Immunological Assays to Screen for Ricin-Containing Samples. Toxins.

[10] He X., Patfield S., Cheng L.W., Stanker L.H., Rasooly R., McKeon T.A., Zhang Y., Brandon D.L. (2017). Detection of Abrin Holotoxin Using Novel Monoclonal Antibodies. Toxins.

[11] Worbs S., Kampa B., Skiba M., Hansbauer E.M., Stern D., Volland H., Becher F., Simon S., Dorner M.B., Dorner B.G. (2021). Differentiation, Quantification and Identification of Abrin and Abrus precatorius Agglutinin. Toxins.

[12] Becher F., Duriez E., Volland H., Tabet J.C., Ezan E. (2007). Detection of functional ricin by immunoaffinity and liquid chromatography-tandem mass spectrometry. Anal. Chem..

[13] Wu Y., Taisne C., Mahtal N., Forrester A., Lussignol M., Cintrat J.C., Esclatine A., Gillet D., Barbier J. (2023). Autophagic Degradation Is Involved in Cell Protection against Ricin Toxin. Toxins.

[14] Bevilacqua V.L., Nilles J.M., Rice J.S., Connell T.R., Schenning A.M., Reilly L.M., Durst H.D. (2010). Ricin activity assay by direct analysis in real time mass spectrometry detection of adenine release. Anal. Chem..

[15] Hale M.L. (2001). Microtiter-based assay for evaluating the biological activity of ribosome-inactivating proteins. Pharmacol. Toxicol..

[16] Dawson R.M., Paddle B.M., Alderton M.R. (1999). Characterization of the Asialofetuin microtitre plate-binding assay for evaluating inhibitors of ricin lectin activity. J. Appl. Toxicol..

[17] Brzezinski J.L., Craft D.L. (2007). Evaluation of an in vitro bioassay for the detection of purified ricin and castor bean in beverages and liquid food matrices. J. Food Prot..

[18] Wahome P.G., Bai Y., Neal L.M., Robertus J.D., Mantis N.J. (2010). Identification of small-molecule inhibitors of ricin and shiga toxin using a cell-based high-throughput screen. Toxicon.

[19] Stechmann B., Bai S.K., Gobbo E., Lopez R., Merer G., Pinchard S., Panigai L., Tenza D., Raposo G., Beaumelle B. (2010). Inhibition of retrograde transport protects mice from lethal ricin challenge. Cell.

[20] Pauly D., Worbs S., Kirchner S., Shatohina O., Dorner M.B., Dorner B.G. (2012). Real-time cytotoxicity assay for rapid and sensitive detection of ricin from complex matrices. PLoS ONE.

[21] Oda T., Komatsu N., Muramatsu T. (1997). Cell lysis induced by ricin D and ricin E in various cell lines. Biosci. Biotechnol. Biochem..

[22] Saxena N., Phatak P., Chauhan V. (2022). Differential toxicity of abrin in human cell lines of different organ origin. Toxicol. In Vitro.

[23] Rasooly R., He X. (2012). Sensitive bioassay for detection of biologically active ricin in food. J. Food Prot..

[24] Pasetto M., Barison E., Castagna M., Della Cristina P., Anselmi C., Colombatti M. (2012). Reductive activation of type 2 ribosome-inactivating proteins is promoted by transmembrane thioredoxin-related protein. J. Biol. Chem..

[25] Tolleson W.H., Jackson L.S., Triplett O.A., Aluri B., Cappozzo J., Banaszewski K., Chang C.W., Nguyen K.T. (2012). Chemical inactivation of protein toxins on food contact surfaces. J. Agric. Food Chem..

[26] Sowa-Rogozinska N., Sominka H., Nowakowska-Golacka J., Sandvig K., Slominska-Wojewodzka M. (2019). Intracellular Transport and Cytotoxicity of the Protein Toxin Ricin. Toxins.

[27] Makdasi E., Laskar O., Milrot E., Schuster O., Shmaya S., Yitzhaki S. (2019). Whole-Cell Multiparameter Assay for Ricin and Abrin Activity-Based Digital Holographic Microscopy. Toxins.

[28] Sandvig K., Olsnes S., Pihl A. (1976). Kinetics of binding of the toxic lectins abrin and ricin to surface receptors of human cells. J. Biol. Chem..

[29] Leonard J.E., Grothaus C.D., Taetle R. (1988). Ricin binding and protein synthesis inhibition in human hematopoietic cell lines. Blood.

[30] Chan L.N., Li J.S., Liu S.Y. (1985). Differential effects of abrin on normal and tumor cells. J. Cell Physiol..

[31] Nagatsuka T., Uzawa H., Ohsawa I., Seto Y., Nishida Y. (2010). Use of lactose against the deadly biological toxin ricin. ACS Appl. Mater. Interfaces.

[32] Rasooly R., He X., Friedman M. (2012). Milk inhibits the biological activity of ricin. J. Biol. Chem..

[33] Lumor S.E., Deen B.D., Ronningen I., Smith K., Fredrickson N.R., Diez-Gonzalez F., Labuza T.P. (2013). Assessment of the inhibition of ricin toxicity by lactose in milk. J. Food Prot..

[34] Brandon D.L., Adams L.M. (2015). Milk matrix effects on antibody binding analyzed by enzyme-linked immunosorbent assay and biolayer interferometry. J. Agric. Food Chem..

[35] Hegde R., Maiti T.K., Podder S.K. (1991). Purification and characterization of three toxins and two agglutinins from Abrus precatorius seed by using lactamyl-Sepharose affinity chromatography. Anal. Biochem..

[36] Tang J., Xie J., Shao N., Yan Y. (2006). The DNA aptamers that specifically recognize ricin toxin are selected by two in vitro selection methods. Electrophoresis.

[37] Luo L., Yang J., Li Z., Xu H., Guo L., Wang L., Wang Y., Luo L., Wang J., Zhang P. (2022). Label-free differentiation and quantification of ricin, abrin from their agglutinin biotoxins by surface plasmon resonance. Talanta.

[38] Li Z., Xu H., Ma B., Luo L., Guo L., Zhang P., Zhao Y., Wang L., Xie J. (2022). Neutralizing Monoclonal Antibody, mAb 10D8, Is an Effective Detoxicant against Abrin-a Both In Vitro and In Vivo. Toxins.

